# Studies of Geosphere Interactions by Means of Laser Interference Complex

**DOI:** 10.3390/s26020569

**Published:** 2026-01-14

**Authors:** Grigory Dolgikh, Sergey Budrin, Stanislav Dolgikh

**Affiliations:** V.I. Il’ichev Pacific Oceanological Institute, Far Eastern Branch Russian Academy of Sciences, 690041 Vladivostok, Russia; dolgikh@poi.dvo.ru

**Keywords:** laser interference complex, laser strainmeter, laser nanobarograph, microdeformations, supersensitive detector of hydrosphere pressure variations

## Abstract

This paper describes the results of monitoring wave processes in the geospheres using laser interference instruments, a weather station, a seismometer, and other measuring devices. Processing in situ data revealed general patterns in seismic events and variations in the hydrosphere and atmospheric pressure. Laser strainmeters and a seismometer were used to identify natural and anthropogenic seismic activity. A laser nanobarograph and strainmeters allowed us to detect baro-deformation interactions. Processing data from supersensitive detectors of hydrosphere pressure variations, a tide gauge, and temperature sensors revealed regional features of marine wave processes.

## 1. Introduction

The most energy-intensive processes of the Earth occur at the boundary of the system interface of the atmosphere, hydrosphere, and lithosphere. Some of these processes are catastrophic for humanity. Studies of these processes are often conducted on a case-by-case basis, limited to measurements in only one geosphere. However, as recent studies [1,2] show, the primary sources of some wave processes, for example, those occurring in the hydrosphere, can be recorded by instruments located in neighboring geospheres. Thus, microseismic oscillations of the upper layer of the Earth’s crust on the coast can be caused by both wind and swell waves [3,4] and infragravity sea waves [5]. Thus, by measuring oscillations of the upper layer of the Earth’s crust in the coastal zone, we can have a complete understanding of the wave processes developing in the studied water area [6,7]. Using knowledge of such geosphere interactions, we can record sea waves without use of hydrophysical instruments and in areas where their use is limited or impossible, such as in Arctic regions and ice-covered waters. The measurement method described above proved applicable in research on climate change. In [8], results of analysis of data from seismometers at the University of California, Berkeley, for 90 years, from 1931 to 2021, are presented. From the data analysis, the author concluded that there has been a permanent increase in the amplitude of the microseismic background, caused by an increase in the significant wave height, starting from 1970. The author associates this process with anomalous variability in pressure and temperature in the near-surface air layer in the extratropical part of the North Pacific Ocean basin.

Measuring microseismic noise can provide information not only about wave processes, but also about propagation of hazardous atmospheric phenomena that create infrasonic oscillations known in the literature as the “voice of the sea” [9] or microbaroms [10]. Infrasonic oscillations of the upper layer of the Earth’s crust in a range from 5 to 12 Hz, corresponding to the range of the phenomenon described above, have been repeatedly recorded by seismic instruments during passages of extratropical cyclones over the waters of the Sea of Japan [11]. The oscillations were recorded both during cyclones’ passage across the Korean Peninsula and along the coast of the Primorsky Territory.

An interesting and little-studied process of geosphere interaction is meteotsunamis, whose oscillation periods range from 2 min to 2 h and correspond to the periods of seismic tsunamis. According to [12,13], the primary mechanisms of meteotsunami formation are atmospheric disturbances amplified by the resonant properties of coastal topography. Such oscillations are capable of generating sea-level fluctuations in minute ranges (seiches), repeatedly recorded in Japan and the Kuril Islands [14].

To study the patterns of occurrence and development of these processes, it is necessary to apply complex research methods using equipment that allows for measurements of the main parameters of the geospheres with high accuracy in a wide frequency range.

One of the immediate and important tasks addressed by comprehensive research using a wide variety of instruments is continuous monitoring of all geospheres’ parameters and their prompt processing, storage, and cataloguing. A continuous series of experimental data can provide a complete understanding of many physical processes occurring in various environments and help explain the patterns of their origin and development. Such comprehensive measurements are also necessary for studying inter-geosphere interactions and identifying the mechanisms of energy transfer from one geosphere to another. Hazardous geo- and hydrodynamic phenomena are of particular concern, and continuous monitoring of their propagation environment is essential for their study. With the help of this comprehensive approach, we can determine the causes of their occurrence, which can subsequently aid in forecasting and prediction.

## 2. Microdeformations of the Upper Layer of the Earth’s Crust, Registration, and Studies

**Figure 1:**
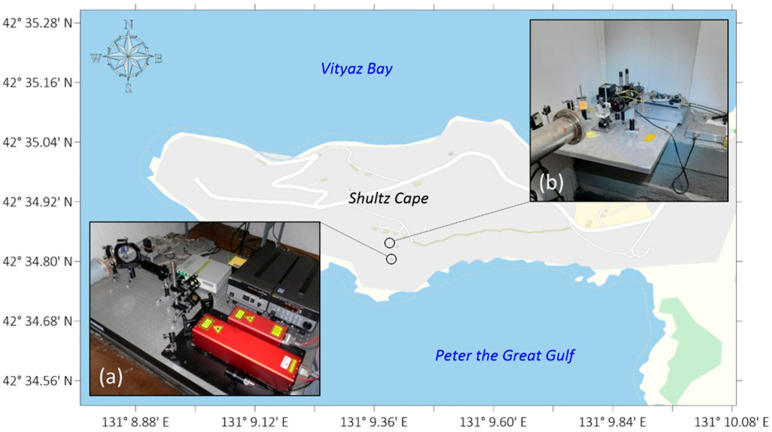
Layout of the orthogonal system of “North-South” (a) and “West-East” (b) laser strainmeters.

“International Scientific and Educational Geosphere Test Site”, located at the “Shultz Cape” Marine Experimental Station (MES), helped us solve all of the above-mentioned tasks, in particular, the continuous monitoring of geosphere parameters. The primary measuring instrument is a system of orthogonal laser strainmeters [15] (Figure 1), constructed based on the optical scheme of an unequal-arm Michelson interferometer. A frequency-stabilized laser from Melles Griot is used as a light source, with a long-term stability of 10^−9^–10^−10^. The entire laser beam path between the reflector and the interference unit is located in a light guide made of polypropylene pipes. The reflector is mounted on an elastic base. The essence of the laser strainmeter measurements consists of using a polarized beam and dividing it into two, passing one of them along the measured arm and the other along the reference arm. The measuring portion of the beam passes along the light guide, hits the reflector, and returns back to the translucent plate. The reference portion of the beam passes through an adjustment unit consisting of mirrors and returns to the translucent plate. When the beams are aligned, an interference pattern appears; changes in it are then evaluated using photoelectronic equipment. The spot with the interference pattern is analyzed using a photodiode and a recording system. Thus, the device can record microdeformations in the Earth’s crust in the frequency range from 0 (conditionally) to 1000 Hz with an accuracy of 0.3 nm over a virtually unlimited dynamic range. The accuracy and measurement error of the interferometer depend directly on the radiation source, namely, on the stability of the laser frequency. Thus, interferometers have an advantage over classical deformation meters [16].

**Figure 2:**
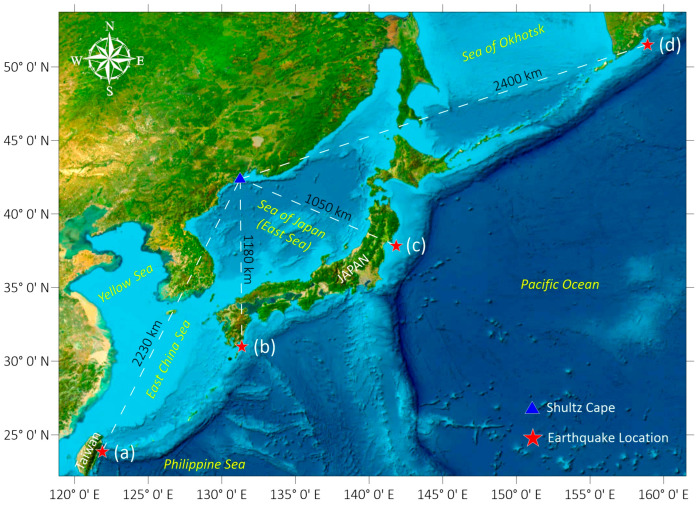
Map of some seismic events recorded by the laser interference system in 2024. (a) Taiwan Island, (b) Kyushu Island, (c) Honshu Island, (d) Kamchatka Peninsula.

This system allows us to study a variety of processes occurring in the upper layer of the Earth’s crust, to assess the impact of variations in atmospheric and hydrosphere pressure, and to study the transformation of acoustic energy into surface seismic waves. One of the most important tasks was recording seismic events of various origins. Numerous earthquakes were recorded in 2024; Figure 2 shows the geography of some of them.

**Figure 3:**
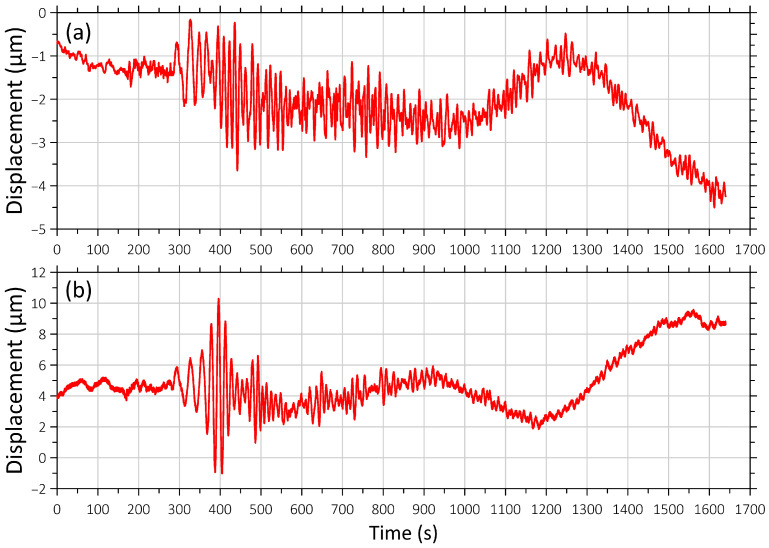
Recording of the earthquake on 8 August 2024 at 07:42:55 (UTC) near the southern coast of Kyushu Island (Figure 1, b). (**a**) Signal from the “West-East” laser strainmeter component; (**b**) signal from the “North-South” laser strainmeter component. The beginning of the recording fragment coincides with the time of the earthquake onset.

**Figure 4:**
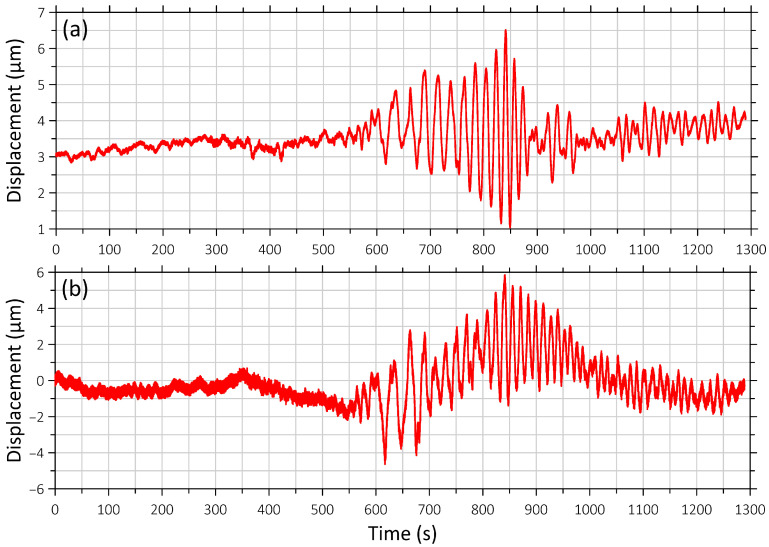
Recording of the earthquake on 17 August 2024 at 19:10:28 (UTC) off the east coast of Kamchatka (Figure 1, d). (**a**) Signal from the “West-East” laser strainmeter component; (**b**) signal from the “North-South” laser strainmeter component. The beginning of the recording fragment coincides with the time of the earthquake onset.

On 8 August 2024, at 07:42 (UTC), an earthquake with a magnitude of 7.1 occurred in the Hyuga Sea off the coast of Miyazaki Prefecture, Kyushu, Japan, 20 km northeast of Nichinan. On 17 August 2024, an earthquake with a magnitude of 7.0 occurred off the coast of Kamchatka. The epicenter was located 108 km southeast of Petropavlovsk-Kamchatsky in the Pacific Ocean. The epicenter was located at a depth of 6 km below the seabed. There was no tsunami threat following the earthquake, and there was no damage or casualties. Figure 3 and Figure 4 show these seismic events’ recordings.

**Figure 5:**
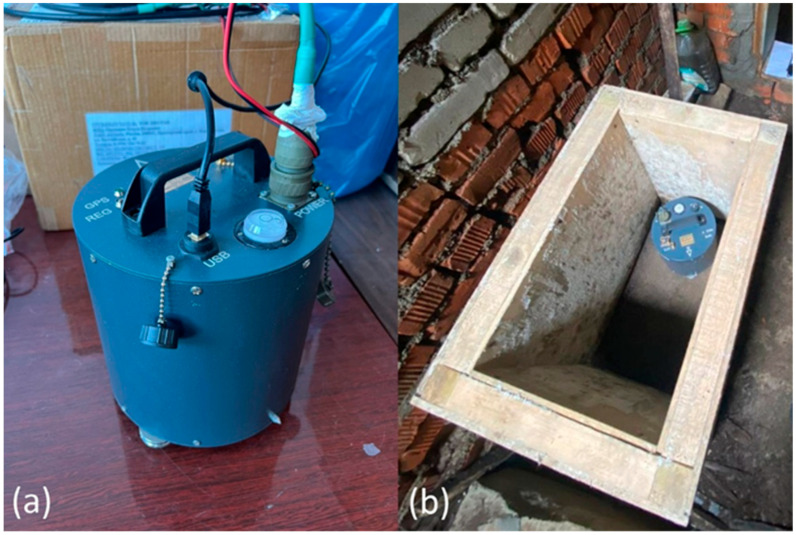
Appearance of the seismometer (**a**); the seismometer installed in a thermally and sound- insulated box in the “West-East” strainmeter component (**b**).

In addition to the system of laser strainmeters for recording seismic events, the laser interference complex includes a seismometer (Figure 5) located in the beam guide gallery of the “West-East” strainmeter component (Figure 1, b).

**Figure 6:**
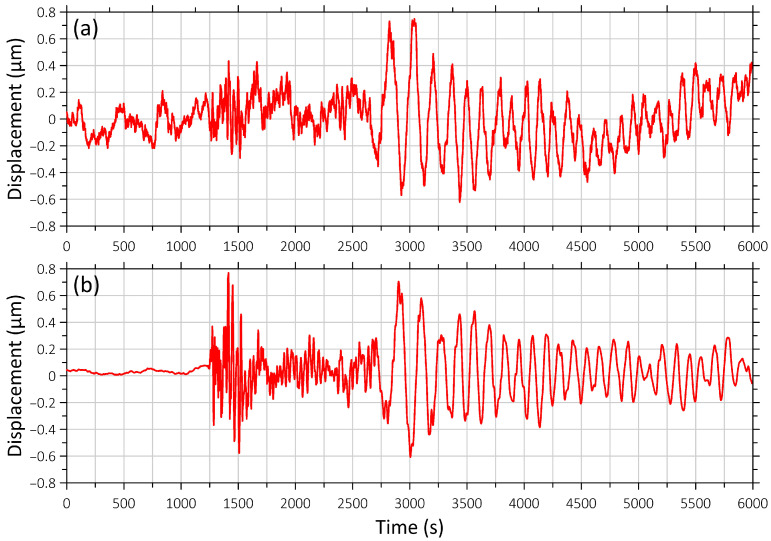
Recording of the earthquake on 4 April 2024 at 03:16:31 (UTC) off the east coast of Honshu Island (Figure 1, b). (**a**) Signal from the “West-East” laser strainmeter component; (**b**) seismometer signal. The beginning of the recording fragment coincides with the time of the earthquake onset.

**Figure 7:**
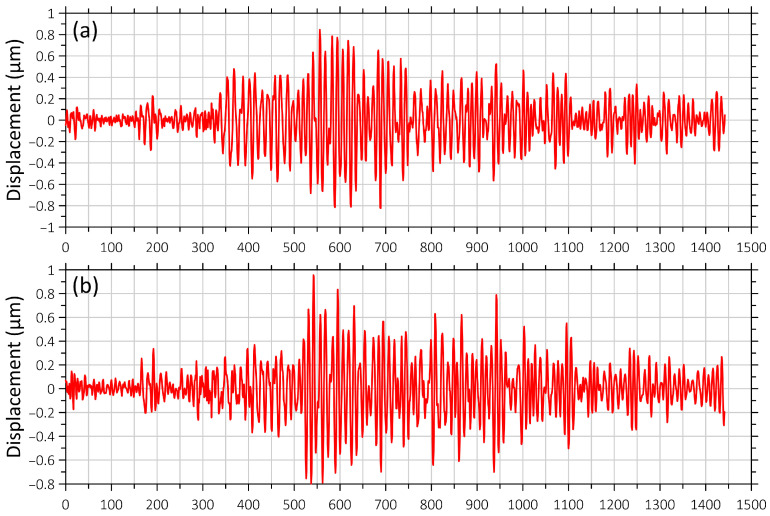
Recording of the earthquake on 22 April 2024 at 18:32:48 (UTC) off the east coast of Taiwan (Figure 1, a). (**a**) Signal from the “West-East” laser strainmeter component; (**b**) seismograph signal. The beginning of the recording fragment coincides with the time of the earthquake onset.

Figure 6 and Figure 7 show synchronous recordings of earthquakes obtained from the seismometer and the “West-East” laser strainmeter component, namely, the earthquake near Honshu Island, which occurred at 03:16:30 on 4 April 2024, at the depth of 29 km, with a magnitude of 6.1; and the earthquake with a magnitude of 6.1 off the coast of Taiwan Island on 22 April 2024, at 18:32:48, at a depth of 6 km (Figure 2, a and c).

## 3. Atmospheric Processes, Registration, and Studies

**Figure 8:**
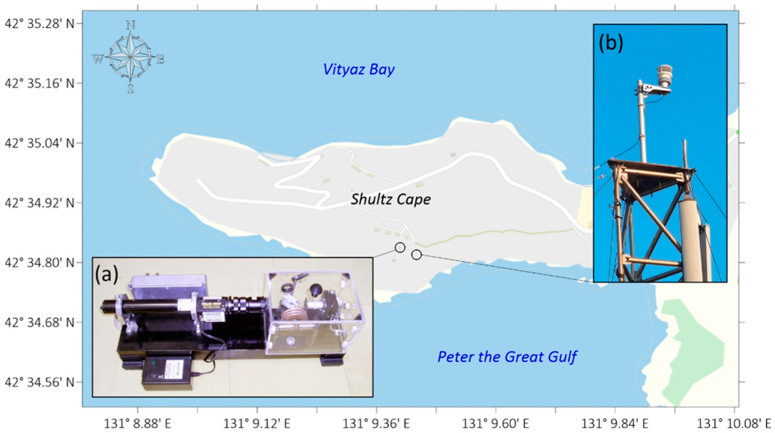
Map of the location of instruments measuring atmospheric parameters: (a) laser nanobarograph and (b) weather station.

One of the areas of ongoing research is the study of the impact of atmospheric phenomena on the upper layer of the Earth’s crust. For this purpose, the “International Scientific and Educational Geosphere Test Site” continuously measures parameters such as pressure, temperature, and wind speed and direction. The instruments and their locations at the test site are shown in Figure 8.

**Figure 9:**
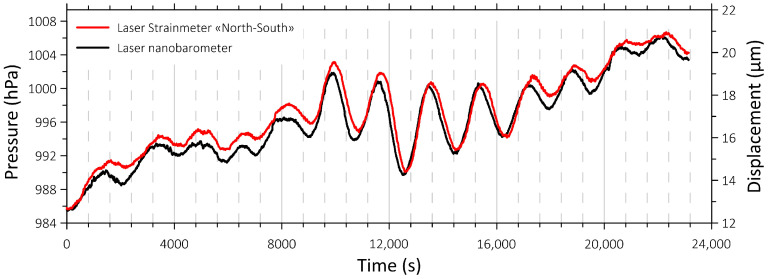
Synchronous recordings by the nanobarograph and “North-South” laser strainmeter component of a registered baro-deformation process. The fragment begins on 23 April 2024 at 20:04:11 (UTC).

A special measuring instrument included in the test site is a laser nanobarograph [17], capable of recording atmospheric pressure variations with an accuracy of 50 μPa. This instrument, compared with synchronous data from laser strainmeters, made it possible to study the baro-deformation interaction between the atmosphere and the upper layer of the Earth’s crust. Figure 9 shows synchronous recordings from the laser nanobarograph and one of the laser strainmeter components.

As Figure 9 shows, both instruments recorded a baro-deformation process with an oscillation period of approximately 3 min, lasting 22 min. Remarkably, the oscillations initially originated in the atmosphere and only after 15 s induced similar oscillations in the upper layer of the Earth’s crust.

**Figure 10:**
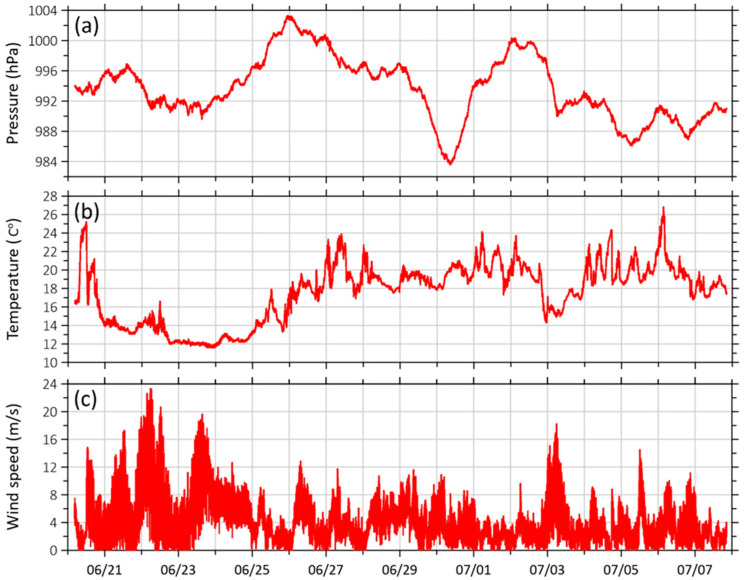
Graphs of variability of (**a**) atmospheric pressure, (**b**) temperature and (**c**) wind speed, obtained from the GMX500 weather station in the period from 20 June 2024 to 7 July 2024.

The main device for recording the parameters of processes developing in the atmosphere is the GMX500 weather station (Gill Instruments Ltd., Lymington, UK); examples of continuous recordings of meteorological data are shown in Figure 10.

**Figure 11:**
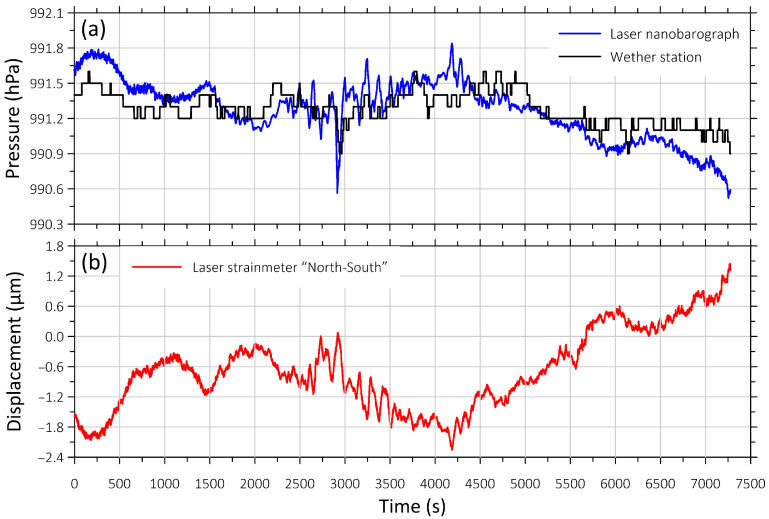
Graphs of variability of atmospheric pressure obtained using the (**a**) laser nanobarograph and the weather station; (**b**) a record of the “North-South” laser strainmeter component obtained during the same period of time. Start of the fragment on 29 July 2024 at 01:33:52 (UTC).

The weather station can effectively record long-term or large-scale processes, such as annual or monthly variations in meteorological parameters and changes in these parameters caused by passages of atmospheric vortices (typhoons). However, it performs poorly on small-scale phenomena due to the limited dynamic range and resolution of the sensor. Figure 11 shows a comparison of recordings from the laser nanobarograph and the weather station.

In Figure 11a we can see that the laser nanobarograph and weather station data are similar in nature and value. However, the weather station pressure graph does not show the 2-min atmospheric oscillations present in the laser nanobarograph recording. Oscillations in the same period were also recorded by one of the components of the laser strainmeter (Figure 11b), indicating an interaction between the atmosphere and the upper layer of the Earth’s crust. Thus, continuous measurements taken by the weather station and laser nanobarograph allow us to determine the origin of certain seismic oscillations in the minute range.

## 4. Hydrophysical Processes, Registration, and Studies

**Figure 12:**
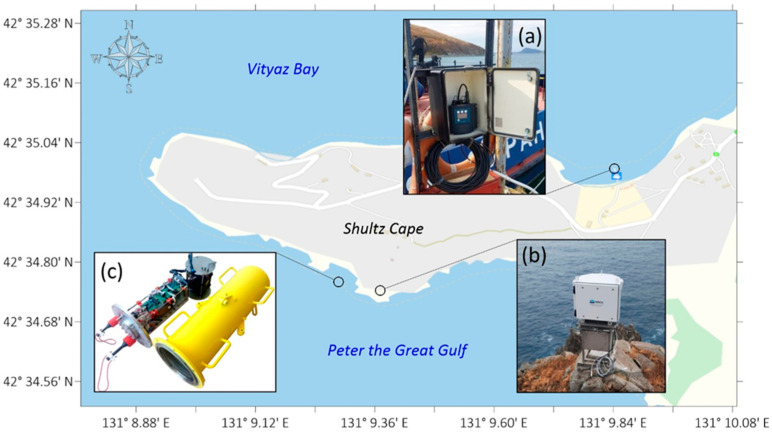
Map of the location of instruments for measuring marine wave processes: (a) coastal tide gauge, (b) radio wave meter, and (c) supersensitive detector of hydrosphere pressure variations.

To study marine wave processes and their interactions with other geospheres, the “International Scientific and Educational Geosphere Test Site” includes instruments for measuring the main parameters of wind and swell waves and also instruments capable of measuring longer-wavelength processes, such as infragravity and internal waves and seiche and tidal oscillations. A map of the instruments’ locations is shown in Figure 12.

**Figure 13:**
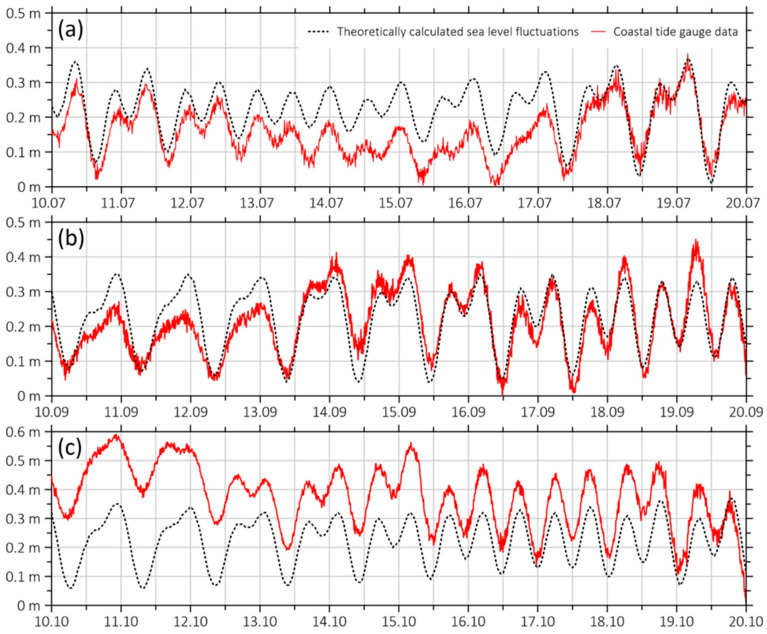
Comparative graphs of experimental data obtained from a coastal tide gauge and theoretically calculated sea-level fluctuations (tides) for the periods (**a**) from 10 July 2024 to 20 July 2024; (**b**) from 10 September 2024 to 20 September 2024, and (**c**) from 10 October 2024 to 20 October 2024.

Continuous measurements of sea-level variations at the “International Scientific and Educational Geosphere Test Site” are conducted using a ValePort TideMaster coastal tide gauge (Figure 12, a) (Valeport Ltd., Totnes, Devon, UK). This instrument is installed on the “Shultz Cape” MES pier; the sensor is submerged 1.5 m below the pier’s edge and firmly secured to one of its abutments. These measurements are necessary to study the impact of long-wave processes (tides) on other geospheres and aid in the interpretation of data obtained from other instruments, particularly in cases of sea-level leap associated with storm up-and-down surges caused by typhoons. With this in mind, sea-level fluctuations are continuously recorded and analyzed by comparing experimental data with theoretically calculated tidal variations. Some comparative graphs are presented in Figure 13.

Regarding tidal characteristics and patterns in all the graphs in Figure 13, we can say that they are identical, differing only in the mean sea level. Looking at Figure 13a, we can see that from July 13th to 16th, there was a decrease in the mean sea level, most likely related to meteorological factors. Figure 13b shows virtually no deviation from the theoretical data, whereas in October (Figure 13c), there was a significant increase in the mean sea level, likely related to the influx of water masses into Vityaz Bay caused by the regular autumn storms and cyclones.

**Figure 14:**
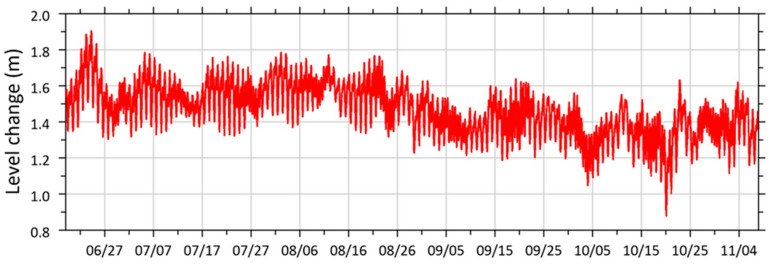
Graph of fluctuations of the mean sea level in the period from 18 June 2024 to 10 November 2024.

Besides analyzing individual fragments of sea-level change recordings, it is crucial to understand the long-term dynamics. Figure 14 shows a continuous recording of sea-level change over several months.

As we can see from the figure above, the mean sea level was decreasing, and while strong fluctuations did occur from September to October, the level itself was lower. Therefore, such an analysis of a continuous series is always necessary when interpreting experimental data.

**Figure 15:**
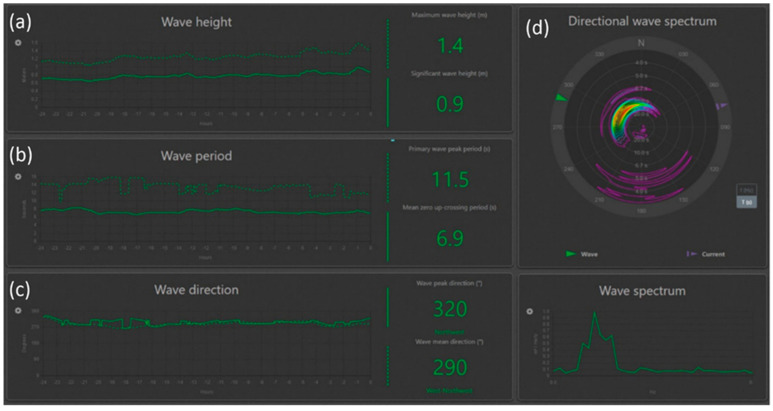
Interface of the radio wave recorder program for operational monitoring of wind wave parameters in a water area. (**a**) Window of wave height data, (**b**) window of wave period data, (**c**) window of wave direction data, (**d**) window of wave spectrum by direction.

The “International Scientific and Educational Geosphere Test Site” also includes a Miros SM-050 wave recorder (Figure 12, b) to obtain real-time information on wind waves and their characteristics. Figure 15 below shows the main wave characteristics recorded by the wave recorder.

All parameters presented in Figure 15 provide comprehensive information about the wave conditions in the water area. This is especially important for installation and retrieval of marine instruments, ensuring their functionality and, consequently, the continuity of measurements. The wave recorder also continuously records wave parameters and stores them on a dedicated server.

The most versatile instruments for measuring wave process characteristics in the study area are supersensitive detectors of hydrosphere pressure variations (Figure 12, c). The main advantage of these instruments is their measuring method. Like the main instruments on the test site, they are based on laser interference measurement methods. Unlike other devices, the sensitive element (reflector) in them is a membrane, which, when acted upon, changes the beam path and, consequently, the interference pattern recorded by the photodetector [18]. Due to their wide dynamic range, they can simultaneously record natural wave processes that vary greatly in frequency and amplitude ranges.

**Figure 16:**
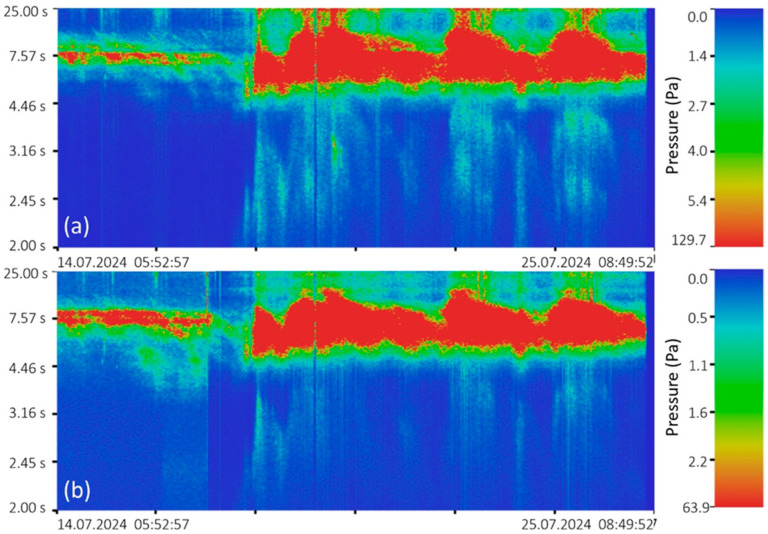
Spectrograms of synchronous recordings from supersensitive detectors of hydrosphere pressure variations from July 14 to 25, 2024. (**a**) Supersensitive detector of hydrosphere pressure variations installed at a depth of 15 m; (**b**) laser supersensitive detector of hydrosphere pressure variations installed at a depth of 25 m.

In 2024, two similar instruments were installed at the “International Scientific and Educational Geosphere Test Site” at different depths in the location shown in Figure 12. Figure 16 shows the spectrograms of the synchronous recording obtained from the instruments.

The spectrograms (Figure 16) clearly show three powerful wave processes occurring one after another. Judging by their duration range of 6 to 10 s and decreasing period over time, these waves came from the open Sea of Japan. It closely matches the data presented in Figure 14, which shows elevated sea levels in July. This suggests that these successive storms likely drove water to the measurement point. Comparing the spectrograms, we also see that local wind waves in the 2- to 4-s range are better recorded by the instrument located at the shallower depth, which is logical. However, the spectrogram (Figure 16b) shows infragravity waves with periods of 20 and 40 s, which are not observed in Figure 16a. This is because depth acts as a natural wave filter. In this case, the pressure field created by lower-amplitude waves with periods of 8–10 s attenuates at depths greater than 15 m, effectively clearing the spectral range from 8 s and above. Because of this, infragravity waves, due to their wavelength and, therefore, energy, become visible on the spectrogram.

**Figure 17:**
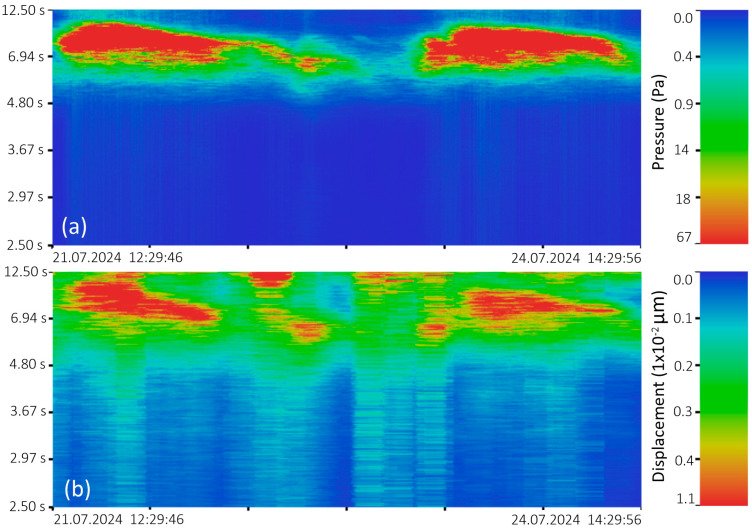
Spectrograms of synchronous instrument recordings for the period from 21 July 2024, to 24 July 2024. (**a**) Supersensitive detector of hydrosphere pressure variations; (**b**) “North-South” laser strainmeter component.

As is well known, marine wave processes can cause micro-displacements in the upper layer of the Earth’s crust [6,8]. Such oscillations are called microseisms or natural earth noise. Figure 17 shows spectrograms of synchronous instrument recordings with registered microseisms.

Figure 17a shows a spectrogram of several wave processes associated with storm conditions over the Sea of Japan. The spectrogram of the laser strainmeter recording (Figure 17b), obtained at the same time, contains microseisms of the first spectral maximum, which are generated by marine progressive waves and are located in the same period range.

**Figure 18:**
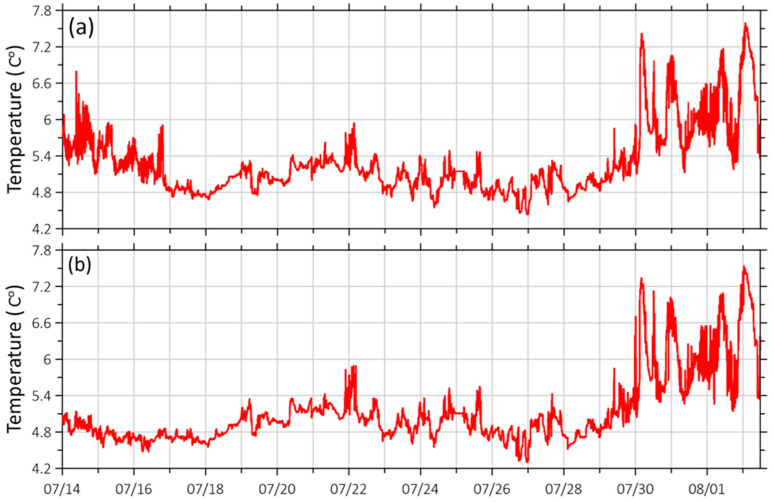
Graphs of variations in bottom temperature obtained in the period from 14 July 2024 to 2 August 2024 from (**a**) depths of 15 to (**b**) 25 m.

In addition to their primary function of measuring wave processes, supersensitive detectors of hydrosphere pressure variations are equipped with temperature sensors and can record both diurnal and seasonal changes in seawater temperature on the bottom. Figure 18 shows fragments of continuous recordings of data from temperature sensors installed on supersensitive detectors of hydrosphere pressure variations.

The graphs in Figure 18 show that temperature differences at each depth vary by only a few degrees. From the 14th to the 18th of June, differences are visible, most likely due to the warming of the water layer and its mixing down to depths of 15 m by the wave process, which we can see in Figure 18. This process is quite weak, has a very narrow range of periods from 8 to 6 s, and is most likely caused by local wave activity. These fluctuations were caused by the fact that temperatures in the 1- to 5-m layer differed significantly from those in the 5- to 10-m layer. As a result, even slight disturbances could cause mixing, but depths below 15 m were unaffected. Between July 18 and 30, temperatures at depths of up to 25 m stabilized. In August, temperatures began to increase at all depths due to heating of the upper layers and their mixing by strong storms and passing typhoons.

**Figure 19:**
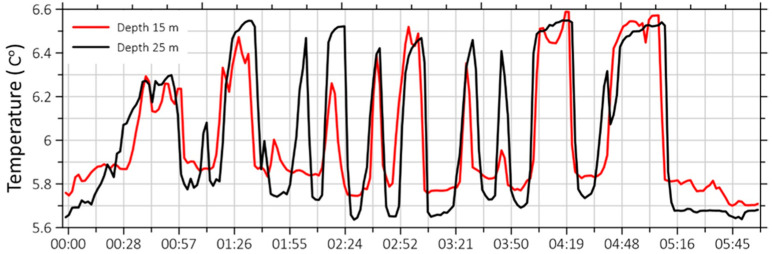
Fragment of temperature fluctuations recording on 1 August 2024.

In addition to large-scale heating and mixing processes, there are also more subtle processes that cause temperature variations near the seabed, such as internal waves. Although large internal waves do not reach the depths at which we conducted measurements, but instead decay at the shelf edge, we can record their “echoes” as temperature fluctuations with periods of approximately 10–20 min. Examples of recordings of this phenomenon are shown in Figure 19.

The oscillation periods in the fragment of the recording shown in Figure 19 range from 15 to 20 min, and their amplitude is no greater than 1 degree. It is obvious that some individual oscillations in the figure attenuate upon reaching a depth of 15 m.

**Figure 20:**
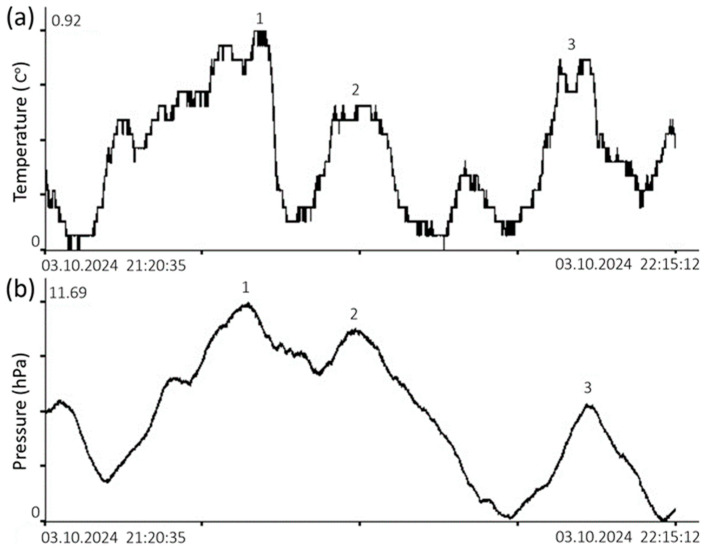
Synchronous records obtained on 3 October 2024, from a (**a**) temperature sensor installed in a supersensitive detector of hydrosphere pressure variations, located on its bottom, and a (**b**) nanobarograph.

However, sometimes temperature fluctuations at the bottom are associated with the interaction of the atmosphere and the hydrosphere, as shown below in Figure 20.

From the figure above, it can be seen that the atmospheric pressure fluctuation of 11.6 hPa, recorded by the nanobarograph, caused bottom temperature fluctuations of almost 1 degree.

## 5. Conclusions

Over several decades of research conducted using laser interference systems, numerous important scientific results concerning inter-geosphere interactions have been obtained. For example, in [19], a numerical estimate of the impact of the pressure field generated by surface sea waves on the upper crust was obtained. Based on this estimate, the coefficient of transformation of sea waves into microseisms of the first spectral maximum and its frequency dependence for the range of wind waves and swell waves were calculated. Numerous results were obtained in the context of studying the mechanisms of transformation of the energy of low-frequency hydroacoustic vibrations into seismic energy [20].

Important results were obtained in the context of climate research. In [21], during the analysis of synchronous experimental data obtained using a nanobarograph, laser strainmeters, and a gas analyzer, a clear correlation was discovered between baro-deformation processes and changes in the concentration of greenhouse gases in the atmosphere.

Some of the most important results were obtained in the study of dangerous geodynamic processes such as earthquakes and tsunamis. In one of the works [22], using the complex presented in this work, the authors discovered an anomalous jump in deformation, recorded by interferometers and occurring during tsunamigenic earthquakes. For example, a similar jump was recorded during a powerful earthquake near the Kamchatka Peninsula on 29 July 2025, which caused a tsunami.

Based on the presented experimental data and their analysis for 2024, using a laser interferometric complex in conjunction with a weather station, seismograph, and other auxiliary equipment, it is possible to solve the following scientific and research tasks:

1. Continuous monitoring of microdeformations processes ongoing in the upper layer of the Earth’s crust, caused by localized processes of seismic activity of natural and anthropogenic nature.
2. Study of phenomena caused by atmospheric processes, which in turn can cause shifts of the upper layer of the Earth’s crust.
3. Registration of microseismic oscillations caused by the passage of cyclones over the waters of the Sea of Japan and determination of their characteristics.
4. Study of the mechanisms of transformation of hydrodynamic energy into the energy of surface seismic oscillations caused by both radiation of low-frequency hydroacoustic signal sources and sources of natural processes.

Solutions to these tasks, through a comprehensive approach to measurements, can help us address several critical issues, namely forecasting and preventing infrastructure damage and harm to human life and health caused by hazardous geo- and hydrodynamic phenomena.

## Data Availability

Third-party data were used. Restrictions apply to the availability of these data. All information about these data and the possibility of obtaining them can be found at chupin@poi.dvo.ru.
